# Pesticides and conservation of large ungulates: Health risk to European bison from plant protection products as a result of crop depredation

**DOI:** 10.1371/journal.pone.0228243

**Published:** 2020-01-30

**Authors:** Daniel Klich, Rafał Łopucki, Anna Stachniuk, Monika Sporek, Emilia Fornal, Marlena Wojciechowska, Wanda Olech

**Affiliations:** 1 Institute of Animal Sciences, Warsaw University of Life Sciences–SGGW, Warsaw, Poland; 2 Centre for Interdisciplinary Research, The John Paul II Catholic University of Lublin, Lublin, Poland; 3 Department of Pathophysiology, Medical University of Lublin, Lublin, Poland; 4 University of Opole, Institute of Biology, Opole, Poland; Sichuan University, CHINA

## Abstract

The coexistence of large mammals and humans in the contemporary landscape is a big challenge for conservationists. Wild ungulates that forage on arable fields are exposed to the negative effects of pesticides, and this problem also applies to protected species for which intoxication by pesticides may pose a health risk and directly affect the effectiveness of conservation efforts. In this paper we assessed the threat posed by pesticides to the European bison *Bison bonasus*, a species successfully restituted after being extinct in the wild. We studied samples of *B*. *bonasus* liver from three free-living populations in Poland (Białowieska, Knyszyńska, and Borecka forests) and captive individuals from breeding centres. LC-QTOF-MS/MS two-step analysis for the detection, identification and confirmation of pesticide residues in liver samples, which included MS and targeted MS/MS scans, was conducted. It was found that European bison are exposed to pesticides as a result of crop depredation: the presence of tetraconazole, fluopyram and diazinon residues in 12 liver samples was confirmed. The concentration levels of the detected substances were quite low, but in the liver samples more than one substance was usually found, and the potential health risk to European bison may result from the synergistic interaction of these substances. The place of occurrence of the population, abundance, and the management regime affect the exposure of European bison to pesticides. Due to the high conservation status of the European bison, the monitoring of intoxication by pesticides should be included in the conservation plans of this species. This issue should also be more widely included in the study of other wild ungulates because knowledge about the impact of pesticides on wildlife is still insufficient.

## Introduction

European bison *Bison bonasus* is an example of the successful restitution of a species extinct in the wild after many years of conservationists' efforts [1, 2, 3]. Population growth is still observed, and in 2017 the total number of individuals in free-living populations exceeded 5,000 [4]. However, the spatial distribution of these populations is unfavourable in most countries of its occurrence. This is due to the fact that European bison occur mainly in large forest complexes, which are not usually spatially connected with each other [5], and the spontaneous colonization of new, suitable areas is rare [6]. As a consequence, some subpopulations of European bison have exceeded the carrying capacities of the forest complexes they inhabit[5, 7], and the measures to minimize this phenomenon have not brought the expected results, or have led to social opposition [8]. For this reason, the management of this species is becoming an increasingly important and difficult challenge for conservationists.

A higher population density may cause increased health risks for the European bison, and may have negative economic consequences for local communities. The main threat is considered to be the transmission of diseases and parasitic invasions to which the European bison is susceptible [9, 10, 11]. One particular danger is bovine tuberculosis, which caused the total elimination of one subpopulation in the Polish Carpathians [12, 5]. Moreover, the increased abundance of the European bison population may lead to considerable damage to forests and crops. Significant damage to tree stands caused by European bison has been noticed [13]. However, the real extent of the damage to forests from this species is not sufficiently known. The extent of the damage to agricultural crops around forest complexes is much better documented. European bison tend to use agricultural crops in locations where the carrying capacity of the forest complexes has been exceeded [14, 15, 16]. An increase in the area of damaged crops as well as compensation costs probably result in a decrease in the acceptance of this species by local communities [8, 17]. However, the home ranges of the studied animals indicate that some of them may partially use field crops (sometimes to a large extent), and some may not use field crops at all [18]. Moreover, the use of agricultural land by European bison depends on the way of management of this species, where lack of feeding inside the forest complex may cause the habit of European bison to extend into field crops [19].

Foraging on arable fields exposes the European bison to contact with plant protection products (pesticides) and may pose a health risk to this species. In Poland, active substances used only for the protection of wheat and rape (the main crops on which the European bison feeds) exceed the number of 130, and new substances are constantly being introduced [20]. The issue of the impact of pesticides on European bison has not been studied so far. At the same time, the results of work on other wild ungulates indicates significant evidence of the adverse effect of pesticides [21]. This includes the under development of the upper facial bones *brachygnathia superior* found to affect, inter alia, white-tailed deer *Odocoileus virginianus*, mule deer *Odocoileus hemionus*, elk *Cervus canadensis*, pronghorn antelope *Antilocapra americana* and bighorn sheep *Ovis canadensis* [21, 22]. These populations were found to suffer from congenital urogenital malformations [23, 24] such as a decrease in penis sheath length, scrotum size, and the change in testes position. Anomalies of reproductive organs are now recognized as being a worldwide problem in an unprecedented number of wild vertebrate species [23]. White-tailed deer *Odocoileus virginianus* were found to suffer from eye deformities, diseases and malformations of the heart and lung, and congenital thymus malformations leading to impairment of the immune system. An increasing number of mammals and birds have been observed with liver tumours, among others wolf *Canis lupus* and rock pigeon *Columba livia* [21].

The aim of the study was to check whether European bison are exposed to pesticides by eating food contaminated with plant protection products, and whether residues of pesticides could be detected in their liver. Secondly, we wanted to estimate the level of concentration of pesticide residues, and consider possible health consequences for the European bison. Thirdly, we wanted to check whether the place of occurrence of the population and its abundance may affect the exposure of European bison to pesticides.

## Methods

### Sample collection

**Fig 1:**
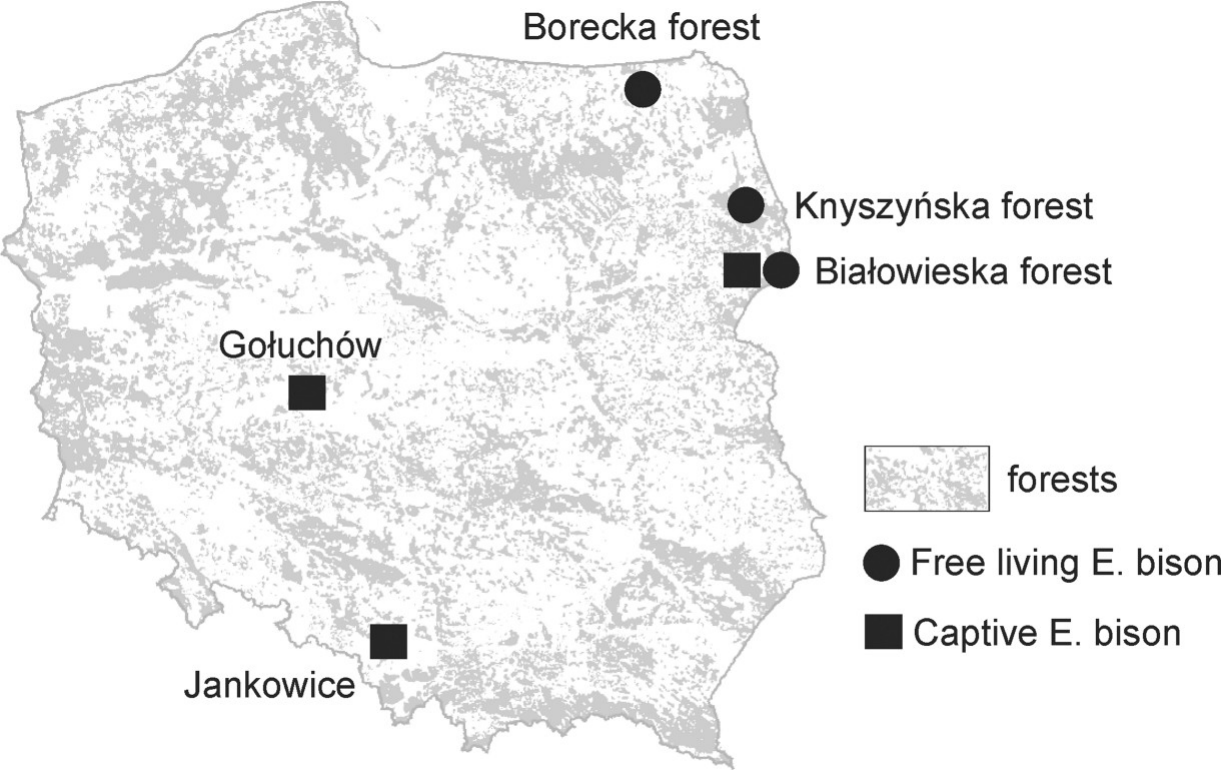
Location of sites where the material (European bison liver samples) was collected.

Our study was based on liver samples collected in the areas of three free-living European bison populations in Poland, located in the Borecka (54°7'20"N, 22°8'30"E), Knyszyńska (53°13'50"N, 23°30'10"E) and Białowieska (52°42'50"N, 23°43'55"E) forests, and in three breeding centres located in Jankowice (50°0'40"N, 19°0'28"E), Gołuchów (51°51'35"N, 17°55'27"E) and Białowieża National Park (52°42'19"N, 23°47'58"E) (Fig 1). All three forest complexes are located in northeastern Poland. In this region currently live about 50% of the individuals of E. bison free ranging in Poland. Knyszyńska and Białowieska forests are adjacent to each other and E. bison migrations are observed between them.

**Fig 2:**
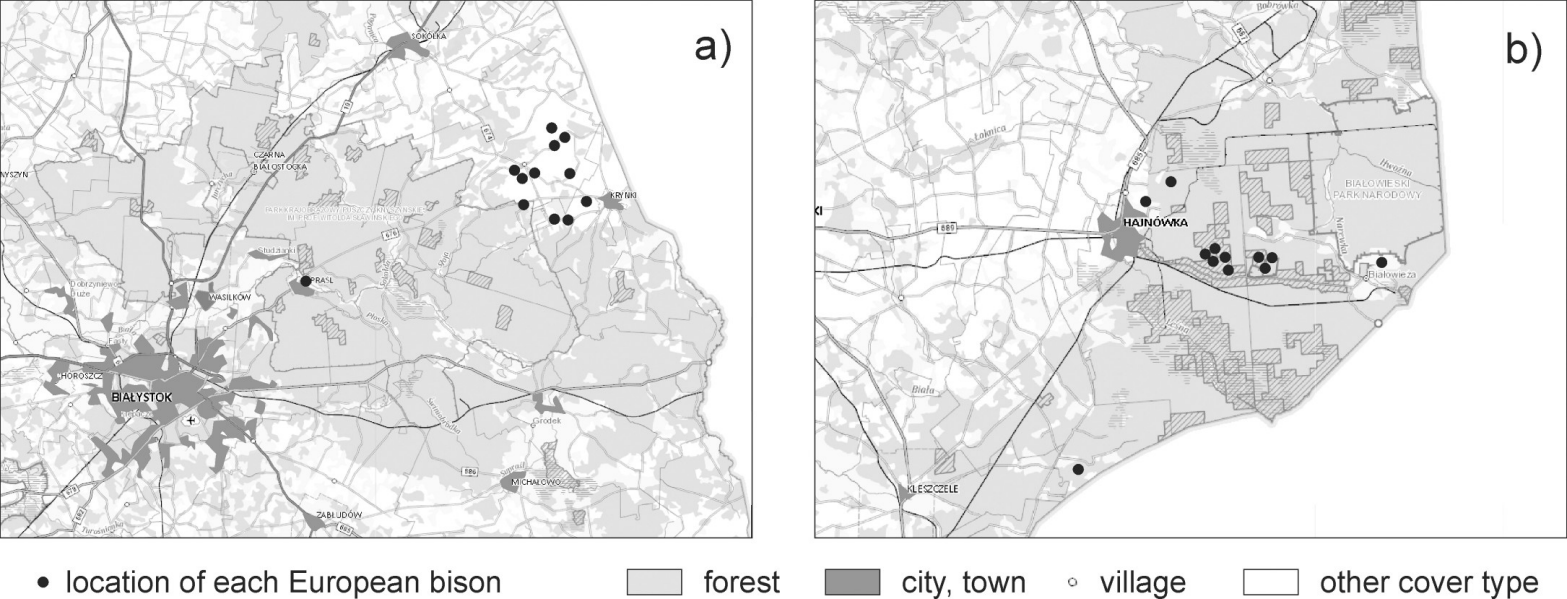
Location of sample collection in Knyszyńska forest (a) and Białowieska forest (b) showing the place of origin of samples in relation to forest and non-forest areas.

In both the Knyszyńska and Białowieska forest complexes frequent cases of crop depredation were observed [14, 15, 16]. However, in the Białowieska forest complex the presence of the European bison on crops is a result of population growth (overpopulation), while in Knyszyńska forest some herds have become used to foraging on field crops as a result of a lack of management in the past [19]. We analyzed the samples from individuals located both within the forest complex and those that were found on crops. In Knyszyńska forest the majority of the samples were collected in agricultural areas, whereas in Białowieska forest they were collected inside the forest complex (Fig 2), which is mainly a result of differences in the lethal control methods used in both areas. Borecka forest presented a forest complex where imperceptible damage is observed in neighbouring crops from this species [16]. Individuals from breeding centres were a control for the presence of pesticides in individuals which are not able to have contact with crop fields in captive conditions. However, the theoretical possibility was given that these individuals could be in contact with pesticide residues in the food provided.

Liver samples were collected post mortem during routine monitoring of dead individuals (found dead or eliminated through lethal control) between 2005 and 2018. The lethal control was carried out by local institutions responsible for European bison management, and each hunter had all necessary permits. Collection and storing of samples of dead individuals for the study was based on the decision of Regional Director of Environmental Protection in Warsaw. According to the decision, collection of dead animals for scientific purposes do not need any permit. The permit without a time limit was given for the storing of the biological samples (including liver samples) of the European bison. All samples were cooled in portable fridge in the field, transported to the laboratory and stored at -20°C until analysis. In total we used 36 liver samples, of which 9 came from Borecka forest, 12 from Knyszyńska forest, 12 from Białowieska forest, and three samples from breeding centres (one from each mentioned above). For each sample, the exact place of death of the individual, sex and age were determined (see Table 1).

**Table 1:** Characteristics of study subjects.

Location (years of obtaining samples)	Sex	Age [years]	Total number of samples
Białowieska forest (2009–2014)	females	20, 22, 25	12
	males	5,5,6,6,9,12,13,16,23	
Knyszyńska forest (2013–2018)	females	4,4,7,10,12,12,16,20	12
	males	3,5,5,8	
Borecka forest (2005–2018)	females	4,5,10,15,16	9
	males	4,4,6,17	
Captive (breeding centres) (2011–2012)	females	4,18	3
	males	13	

### Material and reagents

The high purity (>98%) analytical standards of tetraconanazole, fluopyram and diazinon were purchased from Merck KGaA (Darmstadt, Germany). Stock solution of each pesticide at a concentration of 1000 mg L^-1^ were prepared in acetonitrile and stored at 4°C. The working multicompund standard solutions for matrix-matched calibrations were prepared by appropriate dilution of the stock solutions with acetonitrile. Acetonitrile and methanol (both Optima^®^ LC-MS grade) were pourchased from Fischer Chemical (Waltham, MA, USA). Formic acid (LC-MS grade) and triphenyl phosphate (TPP) were obtained from Merck KGaA (Darmstadt, Germany). Agilent Bond Elut QuEChERS EN Method (buffer salt mixture, polypropylene (PP) tubes and sorbents for dispersive—SPE (dispersive solid phase extraction) including 50 mg primary secondary amine (PSA), 50 mg C18, and 50 mg graphitized carbon black (GCB)) were purchased from Agilent Technologies (Santa Clara, CA, USA). The ultra-pure water was obtained from Millipore Direct-Q3- UV purification system (Merck KGaA, Germany).

### Sample preparation

Pesticide residues were extracted from lyophilised samples using the modified QuEChERS (quick, easy, cheap, effective, rugged and safe) method. One gramme of lyophilized sample was placed in a 50 mL polypropylene (PP) tube. 7.5 mL of miliQ water and 200 μL of 1 μg mL^-1^ TPP (triphenyl phosphate), used as an internal standard, were added. The tube was closed and shaken vigorously. Then 10 mL of acetonitrile was added, and the sample was shaken for 1 min by hand. After this step, the tube was sonicated for 15 minutes in an ultrasonic bath. Then a mixture of 4 g MgSO_4_ anhydrous, 1 g NaCl, 1 g tri-sodium citrate dehydrate and 0.5 g disodium hydrogen citrate sesquihydrate was added, and the tube was again vigorously shaken for 1 min, and centrifuged at 4498 g for 5 min. For the clean-up step, 1 mL of the acetonitrile phase was transferred to a 2 ml centrifuge tube containing 150 mg anhydrous MgSO_4_, 50 mg PSA, 50 mg C18, and 50 mg GCB, and the tube was shaken vigorously for 2 min and centrifuged for 5 min at 12,100 g. Finally, the cleaned extract was transferred into a vial and subjected to LC-QTOF-MS/MS analysis.

### LC-QTOF-MS/MS analysis

The screening and confirmation of pesticides in extracts were carried out using an Agilent 1290 Infinity LC system coupled to a Q-TOF mass spectrometer, model 6550 iFunnel Agilent Technology, equipped with an electrospray Jet Stream working in positive ion mode. Chromatographic separation was achieved using a Zorbax Extend C18 Rapid Resolution analytical column of 2.1×100 mm and 1.8 μm particle size (Agilent Technology) thermostated at 50°C. The mobile phase consisted of 0.1% formic acid in water (A) and 0.1% formic acid in acetonitrile (B); a linear gradient from 5% B to 95% B in 15 min was applied, which was followed by 95% B hold for 3 min, and column conditioning for 4 min. The flow rate was 0.5 mL min^-1^. The MS ion source gas temperature was 250°C and the flow rate was 12 L min^-1^. The capillary voltage was set at 3000 V and the fragmentor at 350V. The MS and MS/MS scanning range was set from 100–1000 m/z and from 20–1000 m/z, respectively. The reference ions of m/z 121.0509 and 922.0098 were used for internal mass correction. Instrument control and data analysis were performed using Agilent MassHunter Workstation Software.

### Screening strategy and pesticide quantitation

Two-step analysis for the detection, identification and confirmation of pesticide residues in liver of European bison, which included MS and targeted MS/MS scans, was conducted. In the first step, the extract of a liver sample was analyzed by LC-QTOF- MS/MS, working in the MS scan. The data were then extracted and screened across an accurate mass database using the Agilent MassHunter Profinder (B.10) software by applying the “Batch molecular future extraction” workflow. The Agilent Pesticide accurate mass database (*Agilent Pesticide LC/MS PCDL*) was used for the retrieval of pesticides that were suspected to be present in the liver sample. Particular attention was paid to these active substances, which are authorized for the cultivation of cereals and rape, as the European bison feeds most often on these two types of crops. The suspected pesticides were then analysed in the second injection in the targeted MS/MS mode at two collision energies, 20 and 40. Identification of compounds was carried out by comparing experimental spectra with a commercial MS/MS spectral library *(Agilent Pesticide LC/MS PCDL*). All detected pesticides in the samples were finally fully confirmed by comparing their MS/MS spectra with the MS/MS spectra obtained experimentally by injection of their analytical standards in the LC-QTOF-MS/MS system. All screening procedures and confirmation of the pesticides’ identity were carried out according to the SANTE European Commission guidelines for pesticide analysis [25]. Confirmation of analytes was based on retention time (± 0.2 min), accurate mass (mass error < 5 ppm, isotopic distribution score > 80%) and MS/MS spectra match (matching score > 70%). Quantification was based on the peak areas obtained by extracting the most abundant product ion (EIC) of each detected pesticide. Quantitative residue analysis was done using the calibration curve prepared by fortifying liver samples, which were checked to be free of pesticide residues beforehand, with pesticides. The limit of quantification (LOQ) was determined as the minimum concentration of analyse for which its extracted ion chromatogram gave a signal to noise ratio of 10 (LOQ = 10 S/N), and it was empirically verified for quantified pesticides by analysing matrix matched standards at these concentration levels.

## Results

**Fig 3:**
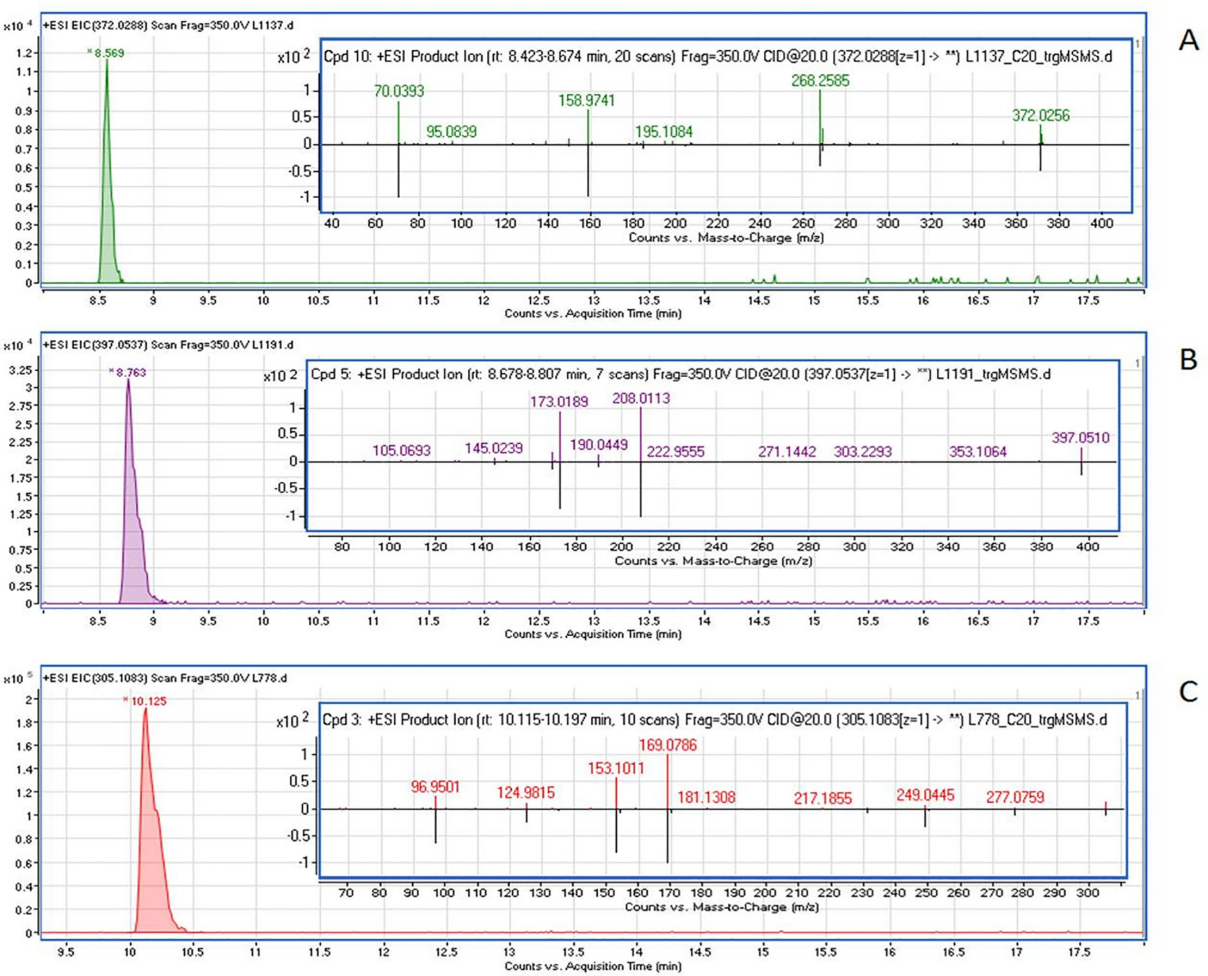
Extracted ion chromatogram of tetraconazole (m/z 372.0288) (A), fluopyram (m/z 397.0537) (B) and diazinon (m/z 305.1083) (C), detected in European bison samples from Knyszyńska forest (female, age 10, 2015; female, age 4, 2016; male, age 8, 2013, respectively) complemented with mirrored MS/MS spectra of the detected pesticide and its analytical standard.

Pesticide residues were found in 12 out of 36 liver samples (Table 2). Three different active substances were identified and confirmed by comparison with spectra acquired for substance standards (Fig 3, Table 2). They were two fungicides (tetraconazole C_13_H_11_Cl_2_F_4_N_3_O and fluopyram C_16_H_11_ClF_6_N_2_O) and one insecticide (diazinon C_12_H_21_N_2_O_3_PS) (Fig 3). In most liver samples in which pesticide residues were found, more than one substance was usually detected (Table 2). Tetraconazole was the most common substance (found in 12 individuals), followed by fluopyram (nine individuals) and diazinon (one individual).

**Table 2:** Concentration of pesticide residues in European bison livers (μg/kg dry mass) in Knyszyńska forest.

Sex	Age (years)	Fluopyram (LOQ = 4 μg/kg)	Tetraconazole (LOQ = 6μg/kg)	Diazinon (LOQ = 4μg/kg)	Year
Male	8	-	3.02*	31.77	2013
Female	10	20.81	10.44	-	2015
Female	12	8.13	2.25*	-	2015
Female	12	4.69	2.37*	-	2015
Male	5	4.58	5.85*	-	2016
Female	20	5.35	3.50*	-	2016
Female	4	3.55*	5.50*	-	2016
Female	4	14.63	3.96*	-	2016
Male	5	-	2.57*	-	2018
Female	7	-	2.31*	-	2018
Male	3	3.68*	4.34*	-	2018
Female	16	5.25	3.50*	-	2018

*below LOQ value

The concentration of identified pesticide residues was generally low. In the case of tetraconazole, for 11 samples the concentrations were below the analytical limit of quantification (LOQ), and its presence could only be confirmed qualitatively. Only for one sample (the liver of a 10-year-old European bison female) was the concentration of tetraconazole higher than LOQ, and was equal to 10.44 μg/kg dry mass. The concentration of fluopyram in the liver samples was also at low levels: two samples below the LOQ, other individuals from 4.58 to 20.81 μg/kg dry mass. In the case of diazinon the concentration was equal to 31.77 μg/kg dry mass (Table 2).

All cases of pesticide residues in the livers came from individuals from the Knyszyńska forest population. In Białowieska forest, where significant crop depredation was also observed, the pesticides were not stated. The population in Borecka forest, as well as control individuals in captivity, were also free from pesticide residues.

Pesticides were detected in 12 individuals, and in many cases values below the LOQ level were obtained. For this reason it was not possible to reliably determine the impact of the sex and age of individuals on the concentration of plant protection product residues in liver.

## Discussion

### Exposure of European bison to pesticides and the need to monitor this phenomenon

Our study has confirmed that residues of active substances of plant protection products can be detected in the liver of European bison from free-living populations (i.e. European bison are exposed to pesticides as a result of crop depredation). The applied analytical method (High Resolution Mass Spectrometry) with the application of both non-targeted and targeted analysis allowed us to carry out screening tests (to indicate a potential list of pesticides found in the samples), and then unequivocally confirm and quantify pesticide residues. The effectiveness of the analyses carried out (including obtaining quantification limits complying with EU regulations) allows us to state that the procedure used in this work could become a method of choice in screening for the presence of pesticides in liver samples of European bison or other wild ungulates.

In further research, one could consider supplementing our procedure with analyses performed with the use of Low Resolution Mass Spectrometry (e.g., triple quadrupole mass analyzers, QQQ) working in multiple reaction monitoring (MRM) mode, which would enable more sensitive detection of toxins in the liver of studied animals, including residues of pesticides found in trace amounts. Detection of traces of toxins is justified because it is known that some compounds can accumulate in the body, and early detection can prevent excessive accumulation, thus reducing the animal's health risk. Although there are very little data on the bioaccumulation and biotransformation of current-use pesticides, such data is critical in assessing their fate and potential toxic effects in wild ungulates. Processes such as bioconcentration and bioaccumulation, and direct impact of toxic substances on populations may lead to changes in the structure of biocenosis [26]. Also worth noting is the synergism of the active substance [27]. Toxic dependence of one substance from the concentration level of another does not have to be linear, and the increase of toxicity can be multiple [28]. In the preparations there is rarely one single active substance. Most frequently these are combinations of two, three or even multiple combinations of several compounds, which can reinforce or multiply their effect. Tests showed that despite the low toxicity of individual components of the mixture, their combined effect can be different [29], and sometimes extremely toxic [30].

### The level of concentration of pesticide residues, their co-occurrence, and possible health consequences for the European bison

The concentration levels of each substance were quite low, and the assessment of the potential risk to the health of the European bison is difficult. In the majority of tested samples of the liver of the European bison, where pesticides were detected, more than one substance was found, and we can assume that their interaction can be synergistic in nature [31].

In nine of the tested individuals two active substances (tetraconazole and fluopyram) belonging to fungicides were detected. Tetraconazole, belonging to triazol, is a group of compounds commonly used in agriculture and medicine. They are used for seed spraying and coating. In Poland there are currently 10 registered fungicides with tetraconazole as the active substance. This substance is a widely used fungicide for wheat and beetroot. Tetraconazole was classified as likely to be carcinogenic to humans based on the occurrence of liver tumours in male and female mice [32]. *In vivo* and *in vitro* tests on rats provide evidence that triazole (i.a. flusilazole) is responsible for causing malfunctions of the development of human foetuses, and adverse effects on heart development and the maxillofacial skeleton [33], resulting in changes to throat anatomy and confluence of heart aortic arch [34]. They affect the process of spermatogenesis in rats [35] and cause infertility in male partridges [36]. Furthermore, in studies conducted on the honey bee (*Apis mellifera*) synergistic toxicity of tetraconazole and other pesticide—imidacloprid, was detected. As a result of using this mixture, the honey bee’s mortality increased by 20%, compared to the added mortality caused by these pesticides when used separately [27].

Fluopyram is a broad spectrum fungicide targeting pathogenic plant fungi (e.g. white dot, black mould, botrytis) [37]. In Poland, it was approved for use in 2014. During the general toxicity evaluation of fluopyram in rodents, the liver was identified as a target organ (hepatomegaly and liver hypertrophy were observed in all studies). At the end of the guideline carcinogenicity study, an increased incidence of hepatocellular adenomas and carcinomas was observed in female rats following exposure to the highest fluopyram dose evaluated (1500 ppm; [37]). A combined fungicide containing fluopyram and tebuconazole was classified as extremely toxic to the earthworm *Eisenia andrei*, especially due to longer retention in the cells [30]. Besides, the latest studies show that combination of these two substances presents genotoxic and cytotoxic potential on rat bone marrow [38].

Another active substance detected in European bison is diazinon, which is a contact organophosphorus insecticide with a wide range of insecticidal activity. It is also available in mixed formulations with other insecticides. Presently, diazinon is not listed on the national insecticide register for products approved for use in Poland. Diazinon may be absorbed from the gastrointestinal tract, through intact skin, and following inhalation. In the dog and guinea-pig, diazinon has been reported to cause acute pancreatitis [32]. Tests of systemic toxicity and developmental neurotoxicity conducted on baby rats showed that the maximum tolerated dose of diazinon is 1–5 mg/kg. Below the maximum tolerated dose, diazinon impaired neurotic outgrowth in the forebrain and brainstem. Diazinon also decreased choline acetyltransferase activity [39]. These tests prove that the low levels of residue of the active substance cannot be disregarded. In our study carried out on European bison, in the liver of only one individual was the active substance with a concentration of 0.03 mg/kg detected. Regarding as a benchmark the transformation of diazinon in sheep, it could be concluded that in the fat tissue of European bison the content of diazinon might be 35 times more than in the liver ([40] after [41]).

### Factors affecting the exposure of bison to pesticides

The place of occurrence of the population, abundance, and the way of management of European bison seem to be the main factors that increase crop depredation and affect exposure to pesticides. We found no pesticide residues in captive or free ranging individuals in Borecka forest. This suggests that this population was not at risk from plant protection products, even if they are intensively fed. Apart from natural fodder, which consists of grasses and parts of woody plants, European bison are also fed with agricultural products. The basic products used in feeding are root crops (beets, carrots, potatoes, swede), as well as corn and cereals, including in the form of silage [42]. Therefore, toxic substances can also get into the European bison from agricultural products. A study [43] showed residues of active substances in root vegetable and potatoes. Eight active substances were detected, and residues of pesticides were detected in 62% of samples of root vegetables and in 4% of potato samples. Other tests carried out on grains (rye, wheat), vegetables (potato, carrots, cucumbers) and fruits also showed the presence of pesticides [44]. However, our results show that the fodder given to European bison, in captive as well as in free-living conditions, is not dangerous for this species compared to foraging on crops, e.g. soon after pesticide application.

In contrast to the population in Borecka forest, we found the presence of pesticides in all tested individuals from Knyszyńska forest. However, in samples from Białowieska forest no pesticide residues were found. This was surprising because in both forest complexes European bison tend to use neighbouring crops, where they feed on cereals and rape [14, 16]. Nevertheless, liver samples were collected from European bison in different locations. In Białowieska forest almost all tested individuals were located inside the forest complex, while the individuals from Knyszyńska forest were located outside the forest complex, on agricultural crops (Fig 2). The home range area of European bison depends on many environmental factors, but is up to several dozen square kilometers [45]. The home range area of some herds can therefore be completely covered within the forest complex, while the home ranges of other herds may to a greater or lesser extent include arable land [18]. It is possible that the specimens from Białowieska forest belonged to herds ranging inside the forest complex, or that their use of agricultural cops is insignificant. However, herds in this forest complex that frequently use crops are probably also exposed to pesticides. Therefore, it can be stated that there may be a risk of exposure of the European bison to pesticides in forest complexes with significant crop depredation, but only for some of the herds that more often use the agricultural landscape.

## Conclusions and future directions

The coexistence of large mammals and humans in the contemporary landscape is a big challenge for conservationists [46]. The example of European bison shows that even a successful restitution and a satisfactory increase in the size of the population may give rise to many new problems and challenges. We show that the growing population, naturally looking for a new food base, may be exposed to uncontrolled intoxication with pesticides used in agriculture which pose the threat of acute poisoning, as well as the accumulation of toxic compounds in the body. Due to the high conservation status of the European bison, this threat should be included in the conservation plans of this species. This applies to both activities to monitor this phenomenon and its health consequences for the European bison. Such monitoring should be tied to health monitoring and be provided in areas of overpopulated populations, especially in herds using field crops as supplementary winter foraging sites. Preventive measures to limit the population density of European bison where crop depredation by this species shows an upward trend should also be undertaken. Failure to inhibit uncontrolled population growth and the spread of herds to arable land could create a threat to the population that is difficult to predict.
